# Hospital and Institutional News

**Published:** 1915-10-02

**Authors:** 


### HOSPITAL AND INSTITUTIONAL NEWS.

### A GIFT TO DEWSBURY INFIRMARY.

At a recent meeting of the Dewsbury Infirmary Board the President made the pleasing announcement of a gift of £1,000 for the endowment of a bed. The donors are the widow and members of the family of the late Mr. Henry Chadwick, who for several years was a member of the Board and to perpetuate whose memory the gift has been made. This donation comes as a very welcome contribution to the funds of the infirmary and is a testimony to the generosity of the citizens of the district. During the time the late Mr. Chadwick was a member of the finance committee he was notably zealous in the performance of his duties, and it must be a pleasing experience for those of his old colleagues who are still on the Board to have this reminder of a former co-worker.

### CENSORING THE GERMAN MEDICAL PRESS.

The military censoring of German medical journals which has for some time been very strict has now been made much more severe, and the Editor of the *Deutsche medizinische Wochenschrift* has requested all contributors to its columns to place their communications before the proper military or medical authorities before they are sent in for publication. This step has been taken because papers have been published which, according to the official view, contained untrue and inaccurate statements and tended to breed uneasiness among the populace. It is also alleged that articles in the German medical Press were being exploited in enemy countries to the detriment of the army. It is hardly necessary to draw upon medical journals for facts detrimental to the German army.

### NO ROOM FOR SLACKERS.

A new light is thrown on the recent official statement that the War Office would be unwilling to suggest that junior medical students should be discouraged from taking combatant commissions by some observations in the current issue of *Guy's Hospital Gazette*. During the last six months it has occasionally been brought to the notice of our contemporary that men have tried to enter the medical profession, not with the idea of showing any enthusiasm for their work, or of ever getting qualified, but with a view to safeguarding themselves should conscription be established in England. Had the War Office some such possibility in view when it refused to discourage junior students from taking combatant commissions? This course of action alluded to in the *Gazette* is very properly described as despicable, and we are glad to learn that Guy's has no use for such men and that no pains have been spared to keep them outside the gates.

### THE PROBLEM OF THE INSURED TUBERCULOUS PATIENT.

Insured persons, when they first come under medical treatment for symptoms suggestive of tuberculosis, are supplied with medicines which are charged to the medical benefit fund and not to the sanatorium benefit fund. At a recent meeting of the Northumberland Insurance Committee a recommendation was made by the sanatorium benefit sub-committee that the doctors should be urged to persuade those of their patients suffering with tuberculosis to apply as soon as possible for sanatorium benefit in order that the drugs and supplies required for their treatment might be charged to the sanatorium benefit fund. Tuberculous patients as a rule are somewhat expensive to treat and their requirements are fairly continuous, and hence unless the above recommendation is duly carried out the medical benefit fund of any given locality is likely to be unnecessarily burdened by expenses which rightly should be borne by the sanatorium benefit fund.

### LONG VOYAGES AND THE FOOD SUPPLY.

In view of the longer voyages of trading vessels due to the war, much food is reaching London in a damaged condition, and those engaged in examining the importations have heavy burdens thrown upon them at the present time. At the wharves in Tooley Street, the egg market of this country, there are vast quantities of food arriving which can never be put on the market, and this to some degree accounts for the high prices now demanded for certain articles, and for the shortage of supplies. Bermondsey has been surfeited with the odour arising from bad eggs which have been destroyed, and grave complaints have been made of the nuisance. In six weeks some millions, representing a tonnage of over 118 tons, have had to be done away with. Few people can realise the great number of eggs represented by this weight, and the frightful waste, which would not be necessary in normal times. Meat, fruit, tinned goods, cheese, potatoes, and bananas have also been condemned in large quantities as unfit for human consumption. Probably there are ways and means of saving some of these goods, but with the long sea voyages trading ships are now forced to take it seems almost hopeless to prevent in any great degree the decomposition of foodstuffs on their way to this country.

### THE CONSUMPTIVE RECRUIT.

In a leading article in The Hospital of October 17, 1914, we drew attention to a report made by Dr. Symms, the medical officer of health for Bath, upon the number of tuberculous men who had been accepted as recruits for the Army. We return to the matter because one of the Leicester Guardians recently accused the medical officer of incompetence because three men certified by him as being consumptive had been accepted for the Army. In a report on the subject to the Guardians the medical officer easily refuted this charge, and pointed out that many cases of undoubted phthisis had been passed for service in the Army and had soon after been invalided out as medically unfit. He added that he knew of other cases which had improved considerably after joining the Army, especially during the South African War. He expressed the opinion that the treatment of phthisis is often unsuccessful because the sufferers refuse to be properly treated and will not perform the graduated work which is essential for their recovery. The point is important, and it would be interesting to have the opinion of other medical officers of sanatoria as to the extent to which it is true.

### THE CLOSING OF POOR-LAW INSTITUTIONS.

The movement towards the closing of superfluous Poor-Law institutions is progressing. The latest example of activity in this direction comes from the Camelford Union. The number of inmates in the local workhouse is very small, and Mr. Court, the Local Government Board inspector, has advised the Guardians to close the institution and send the inmates to Launceston or some other neighbouring Union. The suggestion met with a great deal of opposition, and the decision was postponed to a subsequent meeting. It was urged that some years ago the Guardians had discussed a similar proposal, but had been advised by the Local Government Board not to close the workhouse. Much has happened since then, and now the central authority and most Poor-Law reformers are agreed that the amalgamation of small Unions and the reduction in the number of institutions would make for economy and greater efficiency. The opposition comes only from Guardians and officials interested in the maintenance of the present state of things. Opposition of this kind should not be allowed to stand in the way, and, if necessary, the central authority should make use of its powers to compel a change wherever it is desirable and overdue.

### THE CONTROL OF MEASLES.

Amateur sanitarians often express surprise at the apparent remissness of medical officers of health in not advocating the compulsory notification of measles. From the *Yorkshire Observer* we learn that a certain gentleman in Leeds takes the occasion of every reported local death from measles to write with commendable perseverance to advocate compulsory isolation for this disease. Notification would, of course, be a necessary prelude to isolation on a large scale, but unfortunately the results that could be expected from such a measure would fall far short from that degree of success which would make the enforcement of notification worth while. Measles is an elusive disease, and the question of its control has received much consideration from the best brains in the sanitary services. Attempts which have been made have only resulted in the accumulation of non-immune material in which one day there occurs an explosive outbreak; much alarm and wild talk follows, but all will be forgotten till the next outbreak. What can be done, and should be done, is to impress on the public the fact that measles is not a disease to be treated casually. The number of deaths from measles is a large one, and due mainly to insufficient care being given to affected children by parents who do not realise the dangerous potentialities of an attack of measles.

### A PLEASING INCIDENT AT KING GEORGE HOSPITAL.

Amid the pain and suffering in the hospitals at the present time there are many incidents that show the kindly and sympathetic feelings that cannot even be dulled under the most adverse circumstances. Such a one is recorded by the British Red Cross Society. Outside King George Hospital, where the gift stores are called upon to find some 60,000 cigarettes a week, a child in search of cigarette pictures was run down. The accident necessitated amputation of the leg, and someone wrote to the manufacturers to ask if they could do anything for the child. They promptly promised to provide the little patient with £1 a week for life on receiving by a given date 10,000 of their cigarette pictures. When but half the number required had been collected, the gift stores heard of the offer. The ward sisters took the matter up with enthusiasm, and the patients helped them with alacrity. More than one patient gave his prized collection—a hundred pictures and more—as a whole, and within three days the balance of 5,000 pictures was collected amongst the patients. Thus the pension of £1 a week for life for the child was secured and the value of warrior patients was demonstrated nobly.

### CHOLERA IN THE GERMAN ARMY.

So far little authentic news concerning the outbreak of cholera in Germany, and the measures that are being taken to combat it, have been available. For such information we have to look to neutral countries, and it is to the Berlin letter to the official organ of the American Medical Association that we are indebted for the following particulars. The appearance of several cases of cholera among soldiers returning home from the Front, and several civilians in the Oder district, led the authorities to increase their efforts to prevent the spread of the disease. Every known measure is being resorted to, especially in regard to water sanitation, and the steps previously taken in the Weichsel district, which proved so successful, are now being duplicated in the East. In the opinion of German medical authorities, who appear to be very optimistic, cholera will not assume any alarming proportions. With regard to other infectious diseases it is stated that similar precautions are being taken, and that the results so far have been good, although several cases of typhus, dysentery, meningitis, and typhoid have occurred both in the East and in the West. So far these cases appear to have been restricted almost entirely to the soldiers, and it remains to be seen whether the precautionary measures that have been adopted will be sufficient to prevent epidemics both in the army and among the civil populations.

### POTIONS FOR PALE PROLETARIANS.

Our attention has been drawn to a half-page advertisement in the *Clarion*, a paper which, to judge by other extracts from it, makes a special claim to address the proletariat. This advertisement may have appeared regularly in the *Clarion*, or may be an innovation; but it is of so abominable a character that we feel sure the editor's attention cannot have been bestowed upon it. It purports to be issued by one who possesses the "highest qualification of the Pharmaceutical Society," and it invites all and sundry to take care of their health and save their money by purchasing remedies for many ailments and illnesses which are set forth at length. In each case a sort of prescription, but with no quantities of the ingredients mentioned, is attached to the malady for which it is recommended; and *Clarion* readers are invited to write for such of these remedies as they may think will suit their complaints. In other words, the patient is invited to diagnose his illness and to treat it himself on the routine stock-bottle principle of the "threepenny doctor." We trust the editor of the *Clarion* will perceive the impropriety of this invitation. If he requires any convincing, let him ask any respectable doctor what disease the prescription audaciously numbered 606 is intended for, and why its diagnosis and treatment should be in expert hands. There are other conditions mentioned in the list in which self-treatment would be just as disastrous to the patient and to the public health as in the instance just given.

### A CURIOUS ADVERTISEMENT.

The following strange announcement appeared in the "Personal" column of the *Times* on September 22: "Private hospital for officers in London, feeling financial strain, will give important post to any lady giving liberal support to same. . . ." The A.M.S. authorities who supervise the numerous private hospitals in the Metropolis may be trusted to take appropriate notice of this very improper advertisement, and we do not presume to advise them what it should be. But surely there is a responsibility also upon the *Times* itself not to print, even in the advertisement columns, such a scandalous piece of mendicity. What the important post, offered in exchange for "liberal support," may be is rather difficult to guess. It might be anything from matron-in-chief downwards; but capacity to fill it seems not thought worthy of investigation, for no hint of any other qualification than that of a big subscription is afforded in the wording of the announcement. There is no need for us to enlarge upon the vicious principle here disclosed, or to explain in detail how and why it is unfair to our gallant wounded: the evil is patent on a moment's reflection, and the moral can be drawn by everyone for himself. A private hospital in financial straits can always close its doors without prejudicing officer patients, and we recommend this course to any which may contemplate following the example set out above.

### FAILURES IN THE PROFESSION.

The Annual Report of the Board of Trade on Bankruptcy, which has recently been issued, shows that last year the number of failures under Bankruptcy and Deeds of Arrangement Acts among "Doctors of medicine, surgeons, etc.," was nineteen, with total liabilities amounting to £15,545. The same number of failures was recorded in the previous year, but the liabilities in 1913 totalled almost precisely double last year's amount. The figures in recent years have been as follows: 1912, thirty-five failures, with total liabilities of £39,923; 1911, twenty-seven failures, with total liabilities of £38,322; 1910, thirty-eight failures, with total liabilities of £55,642. It would appear from these figures that the financial position of the profession has undergone a very satisfactory improvement in recent years. When we consider that last year the number of failures was half that of five years ago, and the amount of the liabilities not much more than a quarter, the natural conclusion is that certain influences must have been at work to make the lot of the struggling practitioner a happier one. There has been, however, a marked reduction of failures in all trades and professions. This decrease appears to be entirely attributable to the war, and the special legislation due to it. Creditors, in the circumstances created by the war, voluntarily refrained from pursuing their remedies in many cases. We hope and, indeed, believe that practitioners are doing better and are prospering increasingly on the whole.

### ARMY MEDICAL SERVICE ORGANISATION.

No one who has discussed with our wounded the condition of things at the Front can doubt that the Army needs more medical men, more especially in the Gallipoli Peninsula. The need is likely to grow greater rather than less. The members of the medical profession are willing to make heavy personal sacrifices to meet this need. But it is only reasonable that the profession should demand in return that the Army should make the best use of the medical men it has already enrolled and should not waste them on work which can be done by other men. In peace time, rightly enough, the surgeons of the R.A.M.C. are required to carry out many duties of a non-medical character. In war time, when the best has to be made of a supply which is very definitely limited, they should be released from all such duties so as to be able to concentrate themselves upon their expert work. This the War Office does not yet seem to have realised.

### HOW DOCTORS ARE WASTED.

In every large military hospital there is a registrar who is always a commissioned officer of the R.A.M.C. His duties are almost entirely non-medical, and could be carried out by any intelligent clerk who had received a few weeks' training in Army forms and procedure. Again, the duties of the officer in charge of the hospital consist very largely of matters of general administration. He is expected to combine the work of a medical superintendent, steward, and accountant. In an emergency like the present the system requires a radical alteration so as to permit him to devote the whole of his time to purely medical duties. A change of the character indicated would add practically two medical men to the staff of every military hospital. Another matter, which one often hears criticised and with justice, is the number of commissioned officers of the R.A.M.C. the War Office is retaining in England. The medical man who joins the Army is a man who has found it possible to place his whole time at the service of the country. He should therefore be employed where whole-time service is essential. If properly organised, practically the whole of the work at home could be done by shifts of part-time workers. The whole of the officers of the R.A.M.C. should be abroad except those whose age or physical condition incapacitates them from active service. It is not fair to expect the medical profession to add further to the sacrifices it has suffered until the War Office has made better use, in the two directions indicated, of the medical men who have already joined the Army.

### MEDICAL DEFENCE IN WAR TIME.

From the effects of the war very few things can escape, and we are not surprised to learn that the work of the Medical Defence Union has felt its influence in many ways. The annual report of this organisation, which was adopted at the annual general meeting of members recently held, shows that last year to the ordinary duties of the Union were added tasks which in times of peace seldom come within its purview. Partnership deeds have had to be reconsidered and revised owing to one or more members of a firm accepting military commissions, and relations in respect thereto have had to be adjusted. Questions of military law and of discipline have required consideration, and much correspondence has been carried out with the authorities in relation to pay and allowances and regulations. Difficulties have arisen owing to civilian practitioners suddenly finding themselves under military law without any previous experience of such, and these have been referred to the Union for adjustment or advice. In one instance, we learn from the report, a court-martial was threatened, but owing to the intervention of the Union this was not resorted to and an amicable settlement was arrived at. Many members of the Council are on military service, and the Council meetings have been brightened by the khaki uniforms worn by these officers.

### PANEL DOCTORS' WORRIES.

Complaints as to the working of the Insurance Act have been many, the chief causes of discontent having included the delayed settlement of accounts for work done, and the withdrawal from practitioners' panels of a large percentage of insured persons claimed to be on military or naval service—even in several instances in practices " where only female patients had been accepted." On these and other matters advice has been sought in numerous cases. The Medical Defence Union has been particularly helpful to some of its members engaged in Insurance practice who had to meet charges of neglect often of a frivolous nature. It has happened that practitioners while working at high pressure have been compelled to attend meetings of Insurance Committees in order to refute allegations of a trivial character, the result being that much valuable time has been wasted by such attendance and the preparation of a defence. We are entirely in accord with the Council's contention that no complaints should be considered by Insurance Committees in the future unless statutory declarations are put in as a preliminary to an inquiry being held. This is the procedure adopted by the General Medical Council and is one which effectively prevents frivolous and vexatious allegations being raised before this authority. It is intolerable that busy practitioners should be compelled to neglect their practice in order to attend before a committee to contradict silly statements based on the tittle-tattle of a patient's neighbours.

### EVENING DINNERS FOR SCHOOL CHILDREN.

To school teachers as a class the medical profession has been accustomed to look with confidence for assistance in their efforts to secure that sound body without which the soundest mind is a feeble and ineffective instrument. Teachers, who are so well placed for the observation of healthy children, should not find it difficult to detect the deviations from the normal which constitute disease more or less serious, and every school medical inspector will endorse the opinions of Dr. Bruce-Bays, M.D. (Lond.), expressed in an address read at the recent conference of Cape teachers. In the course of what was really a lecture on elementary medicine applied to school children, Dr. Bruce-Bays managed to touch upon a wide range of diseases and conditions bearing on school life. In particular he uttered a sensible warning against demanding mental work too soon after a heavy meal. He advocated, in fact, the abolition of the substantial meal in the middle of the day; too often this has to be bolted on account of the distance between home and school. A lighter meal which could be brought by the scholars and eaten at greater leisure is preferable, not only in a climate like that of the Cape, but even in this country. The heavy meal of the day should be taken early in the evening according to the reader of the paper, a precept which could surely be made to fit in with the exigencies of domestic service even in working-class homes, for then the return of the wage-earning father would be also due. Risks of dyspepsia ought thereby to be materially diminished, even if the next recommendation—namely, no evening home-work lessons for children under fifteen, could not be carried out—a matter of some difficulty in these days of strenuous examination work.

### IS CANCER CONTAGIOUS?

To this question Dr. A. D. Thomas, the Medical Officer of Health for Bournemouth, gives an affirmative answer. That the disease is increasing in frequency there is but little doubt, even allowing that the enlargement of the figures is due in part to a better and more careful diagnosis. The nature of cancer, in spite of the patient and ingenious methods applied to its study, remains a mystery, and so long as this is the case, confident means of prevention are impossible. Dr. Thomas is so convinced that there is an element of contagion in the spread of the disease, that he has urged the local health authority to provide facilities for disinfection in every recorded case, and these facilities are now in operation in the borough. His suggestion is that the contagious nature of cancer is overlooked because it is so slow in its operation. There are none of the prompt explosions such as are seen in the epidemics of small-pox, scarlet fever, and measles, but this does not prove that spread by contagion is impossible. It is only in comparatively recent years that tubercle has been recognised as spreading by infection, and the recognition has been due not to the likeness of the disease to those which produce obvious epidemics, but to the detection and study of the causal bacillus. The infection is insidious and gradual, not overt and conspicuous. The same may be true, and perhaps in an exaggerated fashion, of cancer. There has long been a popular belief in what have been called " cancer houses " —that is, houses in which successive inhabitants develop cancer; and some of the most careful students continue to hold to the view that cancer is produced by a parasite germ. Hence it is no extreme suggestion to propose that the disease has some measure of infectivity, and even if this be a mere hypothesis, disinfection would seem to be justified, more particularly as it can do no possible harm.

### FACTORIES FOR CONSUMPTIVE SHOEMAKERS.

An important conference took place in Leicester, on Monday of this week, between Dr. Collis of the Home Office; Professor Leonard Kiel and Dr. Moore, of the Medical Research Committee; and the Council of the National Union of Boot and Shoe Operatives, in relation to the probable causes of consumption in the boot and shoe trade, and the best means to be taken to prevent the spread of the disease. The proceedings were private, but we understand that opinions were freely exchanged on both sides, and that agreement was reached on questions of cleanliness, sanitation, and the ventilation of factories. The Medical Research Committee were also strongly in favour of isolating consumptives in a model factory, but were not in a position to give any promise of help on behalf of the Government in this direction. The conference was both interesting and helpful in many ways, although at present there is little prospect of the Council of the Boot and Shoe Operatives' Union taking action to establish and run a model factory without promise of material assistance from the Government. This is disappointing. We should have thought it possible that a separate sanatorium for consumptive workers in the boot trade—such as is contemplated at Northampton (see The Hospital, September 11, 1915, p. 496)—could have been made self-supporting.

### WILL FREE CHOICE OF DOCTOR HAVE TO GO?

The disturbing influence of the war on the National Health Insurance Service is being felt in many ways, and as time goes on the position will become more and more serious. The almost insatiable but absolutely essential requirements of the Army are depleting medical panels all over the country, and Insurance Committees are confronted by the very difficult problem of ensuring an efficient service and at the same time preserving intact the principle of the " free choice of doctor." But there seems to be little doubt that the operation of that principle may be modified; this would be very unfortunate, but in these days, when much more unfortunate things are happening, the panel doctor and the insured patient must make the best of it. Reports of meetings of Insurance Committees from all over the country show how acute the problem is becoming. What is happening in Carnarvonshire was explained at a meeting of the Insurance Committee for that county the other day. Eighteen panel doctors have left to take up military service. In twelve of these cases the remaining doctors in the localities concerned have agreed to attend to the practice of the panel practitioners, and in those twelve places the panel practice is kept intact, whereas if the people were given a free choice the practice would be taken away and the practitioner penalised for serving his country. That would not be acceptable, and would be subversive of the resolution passed by the Committee to keep intact the panel practice. In two of the remaining cases the partner of the absent practitioner is acting on his behalf. In one case a fully qualified assistant, who was with the doctor before he left, had kept up the practice and attended the insured patients, and in the remaining three cases three doctors out of five left at home have agreed to attend to the practice of the absent practitioners, so that the insured person will have a choice of two doctors. These arrangements appear to be adequate; but what will happen in Carnarvonshire and elsewhere when, as may possibly be the case, hardly a practitioner of military age will be left in civil practice?

### "AN UNFAIR BURDEN."

Another difficulty created by the war is due to the fact that from all Insurance districts large numbers of young men have joined His Majesty's Forces; these represent the healthiest lives, and though when in civil life they paid their insurance contributions regularly, they rarely needed medical attendance. Now that they have gone their contributions cease, and there is so much the less for panel doctors, who nevertheless have just as much work to do. The result is that the undue preponderance of unhealthy lives among those remaining has thrown what is termed by the Medical Benefit Sub-Committee for East Suffolk " an unfair burden on panel practitioners." The insurance doctors in this district are not disposed to ask for higher fees at this time of financial stress, but they have asked the Insurance Committee to bring the position to the notice of the Commissioners in the hope that the question of recompense in some form or other shall not be forgotten when the war is over. The absolute futility of asking the Treasury for any extra grants at the present time was emphasised by Mr. Charles Roberts, M.P., Chairman of the National Insurance Joint Committee, in an address he delivered the other day to the North Riding Insurance Committee. In fact, there is good authority for believing that a Bill to amend the Insurance Acts will shortly be introduced, one of the objects of which will be to effect economy by the abolition of separate National Insurance Commissions and by other means.

### INDUSTRIAL FATIGUE AND ITS CONSEQUENCES.

It has long been felt that a systematic investigation of the question of industrial fatigue should be undertaken; but had it not been for the war and the consequent employment of large armies of workers in the production of munitions, we should have had to wait much longer for the beginning of an inquiry the need for which has long been urged upon the authorities. The Committee which the Government has now appointed to consider and advise on questions of industrial fatigue and other matters affecting the personal health and efficiency of workers in munition factories and workshops will, at the outset, be confronted by various problems, but they will probably find of great service the report by Dr. Stanley Kent on a kindred topic, which has been published this week. According to this interim report, one of the difficulties would appear to be to select efficient tests for determining what stage of physical exhaustion a workman has really reached. In America these questions have been investigated much more systematically than in this country, and the Committee will no doubt be able with advantage to draw upon American experience in this respect. We note that in a letter to the *Times* of September 28 Lord Sydenham draws attention to an important point in connection with this inquiry. At this time of supreme national effort it is, he writes, vital that the conditions of labour should be such as to prevent cumulative fatigue, which may be palliated by spirits, to conserve the workman, and to enable him to give his best to the service of the State without mental or physical deterioration. In the more difficult times which will follow the war the need for increasing economic production, and at the same time for jealously guarding the public health, will be forced upon the nation by inexorable necessity. There can be little doubt, as Lord Sydenham says, that intemperance is largely due to the natural tendency to seek relief from over-fatigue in stimulants, which quickly react upon working efficiency, and thus complete a vicious circle of causation. We look to the new Committee for data and suggestions that will prevent so far as is possible the over-fatigue of workers and its consequences.

### A HINT FOR THE KING'S FUND.

The Metropolitan and Liverpool tables which we publish on page 13, though subject to the disadvantages there indicated, may fulfil a useful purpose by drawing attention to the fact that the Metropolis does not as yet contribute through voluntary contributions anything like a sum proportionate to that which is raised for the voluntary hospitals in several of the larger provincial and Scottish cities and towns. The items on the income side of the accounts of the King's Hospital Fund which are least satisfactory are the two which represent the year's contributions from annual subscriptions and donations. When the King's Fund was established it was estimated that £50,000 a year could readily be obtained from these sources; in fact the actual sum derived from annual subscriptions and donations in 1914 amounted to only £31,248. We consider that immediately the great war is ended a special effort should be made by the authorities of the King's Hospital Fund to raise its annual income from subscriptions and donations to at least £50,000 a year. This, we are confident, could readily be done if proper steps were taken and everything was got ready in advance to bring the matter insistently before the public at the right moment. The reason why the yield in annual subscriptions and donations to the King's Hospital Fund is at present so small is undoubtedly largely due to the desire of its Council and managers not to compete in any way with the individual voluntary hospitals. In this connection it may be mentioned that the percentage of income of the general hospitals from annual subscriptions in 1913 was: in London 10.7 per cent., in the provinces 29.4 per cent., and in Scotland 26 per cent., against a percentage from donations in London of 28.3 per cent., in the provinces of 14 per cent., and in Scotland of 8.7 per cent. These figures have a special interest, as they would seem to indicate a much greater sense of individual responsibility towards the voluntary hospitals on the part of the citizens of provincial and Scottish counties and towns compared with the citizens of London and the county of London. Now that these questions have been raised we hope to receive communications from the residents in large towns throughout the United Kingdom who have interested themselves in the voluntary hospitals and the social well-being of the citizens.

### THIS WEEK'S DRUG MARKET.

One of the noteworthy features of the drug market is the continued steady advance in the price of quinine. Until a few months ago this drug had only been affected by the war to a comparatively small extent, one reason being that large stocks existed in London, while there was a fairly steady flow of supplies of the raw material, cinchona bark. But when it is remembered that a very large proportion of the world's requirements in quinine formerly came from German factories, that in recent months the shipments of bark from Java have materially diminished, while the demand for quinine has substantially increased, it is not difficult to account for the recent advances in price. While it is difficult to form a reliable opinion as to the future of this market, there seems little reason to expect that prices will recede to any considerable extent in the near future, and it would perhaps be advisable to replenish stocks at an early date. American oil of peppermint is likely to be dearer. Cream of tartar and tartaric acid have an upward tendency in price. Quotations for iron and quinine citrate have been raised. Chloral hydrate is dearer. One of the natural consequences of the increased duties on sugar and saccharin is that all preparations containing sugar, such as the various syrups, compound liquorice powder, etc., are correspondingly dearer, and quotations for saccharin have been more than doubled.

